# Seasonality Affects Macroalgal Community Response to Increases in *p*CO_2_

**DOI:** 10.1371/journal.pone.0106520

**Published:** 2014-09-03

**Authors:** Cecilia Baggini, Maria Salomidi, Emanuela Voutsinas, Laura Bray, Eva Krasakopoulou, Jason M. Hall-Spencer

**Affiliations:** 1 Marine Biology and Ecology Research Centre, Plymouth University, Plymouth, United Kingdom; 2 Institute of Oceanography, Hellenic Centre for Marine Research, Anavissos, Attica, Greece; 3 Department of Marine Sciences, University of the Aegean, Lesvos, Greece; The Evergreen State College, United States of America

## Abstract

Ocean acidification is expected to alter marine systems, but there is uncertainty about its effects due to the logistical difficulties of testing its large-scale and long-term effects. Responses of biological communities to increases in carbon dioxide can be assessed at CO_2_ seeps that cause chronic exposure to lower seawater pH over localised areas of seabed. Shifts in macroalgal communities have been described at temperate and tropical pCO_2_ seeps, but temporal and spatial replication of these observations is needed to strengthen confidence our predictions, especially because very few studies have been replicated between seasons. Here we describe the seawater chemistry and seasonal variability of macroalgal communities at CO_2_ seeps off Methana (Aegean Sea). Monitoring from 2011 to 2013 showed that seawater pH decreased to levels predicted for the end of this century at the seep site with no confounding gradients in Total Alkalinity, salinity, temperature or wave exposure. Most nutrient levels were similar along the pH gradient; silicate increased significantly with decreasing pH, but it was not limiting for algal growth at all sites. Metal concentrations in seaweed tissues varied between sites but did not consistently increase with pCO_2_. Our data on the flora are consistent with results from laboratory experiments and observations at Mediterranean CO_2_ seep sites in that benthic communities decreased in calcifying algal cover and increased in brown algal cover with increasing pCO_2_. This differs from the typical macroalgal community response to stress, which is a decrease in perennial brown algae and proliferation of opportunistic green algae. *Cystoseira corniculata* was more abundant in autumn and *Sargassum vulgare* in spring, whereas the articulated coralline alga *Jania rubens* was more abundant at reference sites in autumn. Diversity decreased with increasing CO_2_ regardless of season. Our results show that benthic community responses to ocean acidification are strongly affected by season.

## Introduction

Increasing anthropogenic atmospheric CO_2_ is altering the chemistry of surface seawater worldwide, resulting in ocean acidification. Mean surface ocean pH has already decreased by 0.1 units (a 30% increase in H^+^ concentration) compared to pre-industrial times, and is rapidly decreasing [1]. Studies on the effects of ocean acidification indicate that it will impact a wide array of fundamental biogeochemical and biological processes. Early work on the effects of ocean acidification involved experiments that focused on single species in laboratory conditions, where pH variability was minimised, for periods of up to 18 months [2]. This body of work has rapidly advanced our knowledge of the relative sensitivity of different species, which can be used to formulate hypotheses on responses at the community level, although there is a growing realisation of the need to incorporate natural pH variability and species interactions into ocean acidification research [3], [4].

Interactions between species can cause unpredicted responses to increased levels of *p*CO_2_. For instance, Hale *et al*. [5] report that most invertebrate taxa in a mesocosm experiment responded to increased *p*CO_2_ as expected from single species experiments. Nematodes, however, unexpectedly increased in abundance, probably because of altered species interactions. Community responses to ocean acidification will also depend on indirect effects of carbon dioxide, such as altered animal behaviour [6]. Thus, physiology and ecological niche cannot fully predict a species'susceptibility to environmental changes [7]. Moreover, laboratory and mesocosm experiments are usually too brief to ascertain the effect of increased carbon dioxide on climax communities comprising long-lived organisms [2]. Hypotheses formulated using data from short-term single-species laboratory experiments thus need to be tested in complex communities, ideally in real marine ecosystems [8].

Areas chronically exposed to high *p*CO_2_ can be used to assess long-term community responses to ocean acidification [9], [10]. Hydrothermal seeps with high *p*CO_2_ levels occur worldwide [11], but many CO_2_ seeps also have steep gradients in temperature, salinity, total alkalinity, toxic gases and metals, which could confound the ecological effects of carbon dioxide [12]. In addition, volcanic fluids are often enriched in ammonia, silicate and phosphate [13]. Baseline surveys are therefore needed to check the extent to which vent systems can be used as natural ocean acidification laboratories [14], [15].

Only a few CO_2_ seeps have so far been located that are suitable for use as ocean acidification analogues, namely seeps off Italy [9], Papua-New Guinea [16] and Japan [18]. Studies of these sites have shown that benthic biodiversity decreases as seawater *p*CO_2_ levels increase [10], [19]–[22]. Replication of such studies in a wider range of settings would strengthen the evidence for the ecosystem effects of increasing *p*CO_2_ at the landscape scale. Previous studies found that well-fed individuals are more resilient to ocean acidification [23]; a natural ocean acidification analogue in the Eastern Mediterranean could reveal how marine organisms respond to increased CO_2_ levels in oligotrophic areas. This is of global relevance since nutrient-poor regions are thought to be expanding worldwide due to increased thermal stratification of ocean waters caused by ongoing climate change [24].

Most laboratory experiments into the effects of ocean acidification on macroalgae have focused on calcifying species such as coralline algae and *Halimeda* spp.; responses of brown seaweeds to increased carbon dioxide are poorly known [25], [26], even though they are keystone habitat-forming species in temperate regions worldwide[27]. In addition, many experiments on temperate seaweeds have been performed under constant temperature and light regimes, which are not representative of the daily and seasonal fluctuation these organisms experience in nature [28]. Even when macroalgae are exposed to natural temperature and light fluctuation (e.g. using outdoor mesocosms with continuous seawater pumping), experiments are rarely replicated to encapsulate seasonal responses. Seasonal surveys can easily be made at shallow coastal ocean acidification analogues [22], but have rarely been performed. We therefore have scarce knowledge of how seaweeds may respond to ocean acidification over yearly cycles, even though seasonality heavily influences biological responses to ocean acidification [29].

Temperate marine ecosystems undergo large yearly changes in light and temperature regimes, which indirectly influence other factors important for biological communities such as nutrient levels [30]. In the Mediterranean Sea, these three factors strongly influence macroalgal communities: macroalgal biomass peaks in late spring, and community composition changes among seasons [31]. Specifically, many turf algae disappear and most erect algae decrease in cover during the cold season [32].

Our limited ability to predict community responses of macroalgal communities to ocean acidification, and an overall paucity of research performed on Mediterranean species, add value to studies examining community responses to ocean acidification using CO_2_ vents in the Mediterranean Sea. Results from surveys off Ischia and Vulcano (both in Italy) show how increased carbon dioxide is likely to cause changes in macroalgal communities: as CO_2_ increases coralline algae are replaced by fleshy brown algae such as *Dictyota* spp., *Cystoseira* spp. and *Sargassum vulgare* [22] together with decalcified *Padina pavonica* [33]. This response to increased CO_2_ differs from shifts towards opportunistic macroalgal species such as *Ulva* spp. or mat-forming algae reported in stressed marine benthic ecosystems [34]–[38]; there decreased floral complexity can have detrimental effects on local biodiversity [39] and indirectly affect the abundance of many fish species of commercial importance, such as labrids [25], [40]. Carbon dioxide can be a resource that benefits carbon-limited fleshy algae [22], [36].

The aim of this study was first to determine whether CO_2_ seeps off Methana (Aegean Sea, Greece) were suitable for ocean acidification studies, so we monitored temperature, salinity, pH, Total Alkalinity and the concentrations of heavy metals, hydrogen sulphide and inorganic nutrients (nitrite, nitrate, ammonium, phosphate and silicate). As identifying changes in benthic community composition and abundance in a wide range of environmental conditions is crucial to improve predictions of future ecosystem function, we assessed whether benthic communities changed near the CO_2_ seeps in a manner that could be predicted from previous studies. Since timing can influence biological responses to increased carbon dioxide, from mollusc and coral calcification [41] to change in crops yield [42], we assessed whether responses to ocean acidification were modulated by seasonality.

## Methods

### Study area

Methana is a peninsula on the NE coast of Peloponnese in Greece, located at the western end of the Southern Aegean Volcanic Arc, formed by subduction of the African tectonic plate beneath the Eurasian plate. The last eruption on Methana was in 230 BC, but the area is still hydrothermally active [13]. The CO_2_ seeps studied here are located on the northern part of the peninsula. They appeared shortly after the last volcanic eruption, and thermal baths adjacent to the marine seeps have been used since at least the 1st century AD [43]. Gas emissions at our Methana study site are mainly carbon dioxide, with smaller amounts of nitrogen, carbon monoxide and methane (Table 1). Methane concentrations (17–26 ppm) are much lower than those detected at ocean acidification analogues off Ischia (200–800 ppm [9]), Vulcano (1700 ppm [15]) and Papua New Guinea (87–4360 ppm [16]).

**Table 1:** Composition of gases at Methana seep site.

Date	CO_2_ (ppm)	N_2_ (ppm)	O_2_ (ppm)	CH_4_ (ppm)	CO (ppm)	He (ppm)	H_2_ (ppm)
04/06/2006	991000	10700	<400	26	1.6	<5	<5
23/06/2006	970000	30900	5600	17	1.7	<5	<5

Carbon dioxide accounts for over 90% of the emitted gases, with smaller percentages of nitrogen, oxygen, methane, carbon monoxide, helium and hydrogen (data from [17]).

**Figure 1:**
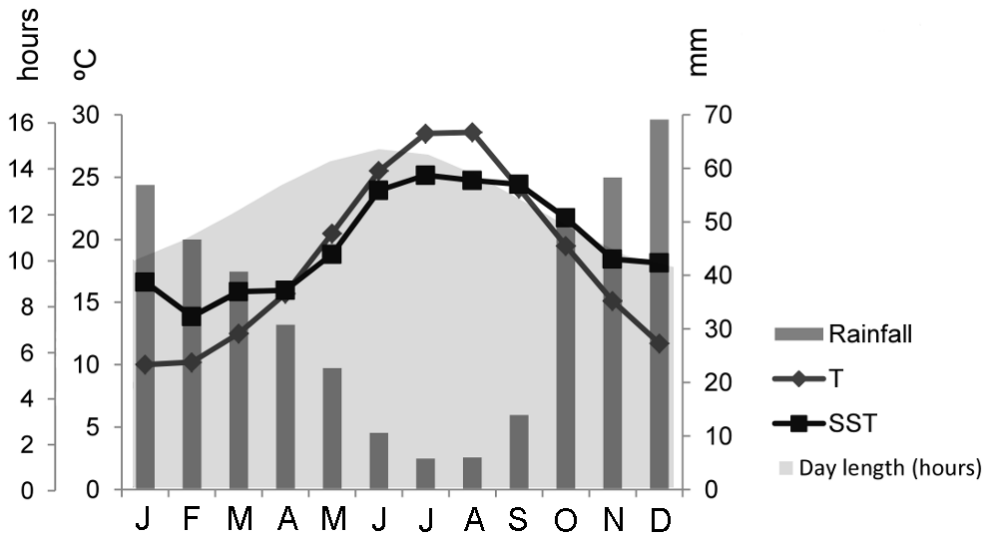
Long-term monthly average day length (hours), rainfall (mm), air temperature (T, °C) and Sea Surface Temperature (°C) for the Saronikos Gulf. SST data are from the World Ocean Atlas 2013 (NOAA), all other data from the World Meteorological Organisation.

The study area is part of the Saronikos Gulf (Central Aegean Sea); this part of Greece is characterised by a Mediterranean climate with strong seasonal differences in temperature, precipitation and day length (Figure 1). The Saronikos Gulf is generally oligotrophic except for its NE part, where wastewater treatment and other anthropogenic pressures along the wider Athens metropolitan coastal front result in increased nutrient loads [44]. Average air temperature varies from 10°C in winter to over 28°C in summer, with sea temperature ranging from 14°C in winter to 25°C in summer. Day length peaks at 14 hours and 43 minutes in June, and is shortest in December (9 hours and 51 minutes).

### Site descriptions

**Figure 2:**
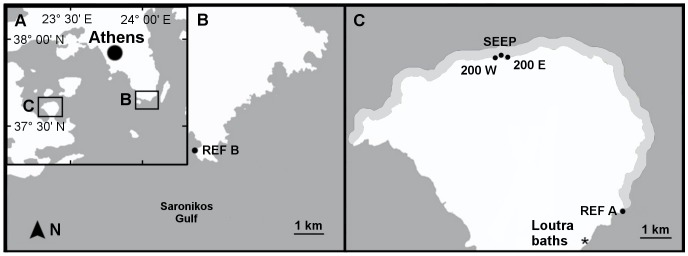
Study sites (points), Loutra baths (*) and area where pH was more variable than at reference sites (light grey). Geographical data downloaded from OpenStreetMap and modified using GNU Image Manipulation Program 2.8.

Preliminary surveys revealed that a small area (∼20 m of shoreline) near the main CO_2_ seeps had a pH_NBS_ constantly below 8.0 (Figure 2), while a much more extensive area had pH variability that exceeded the background conditions of the reference sites.

**Figure 3:**
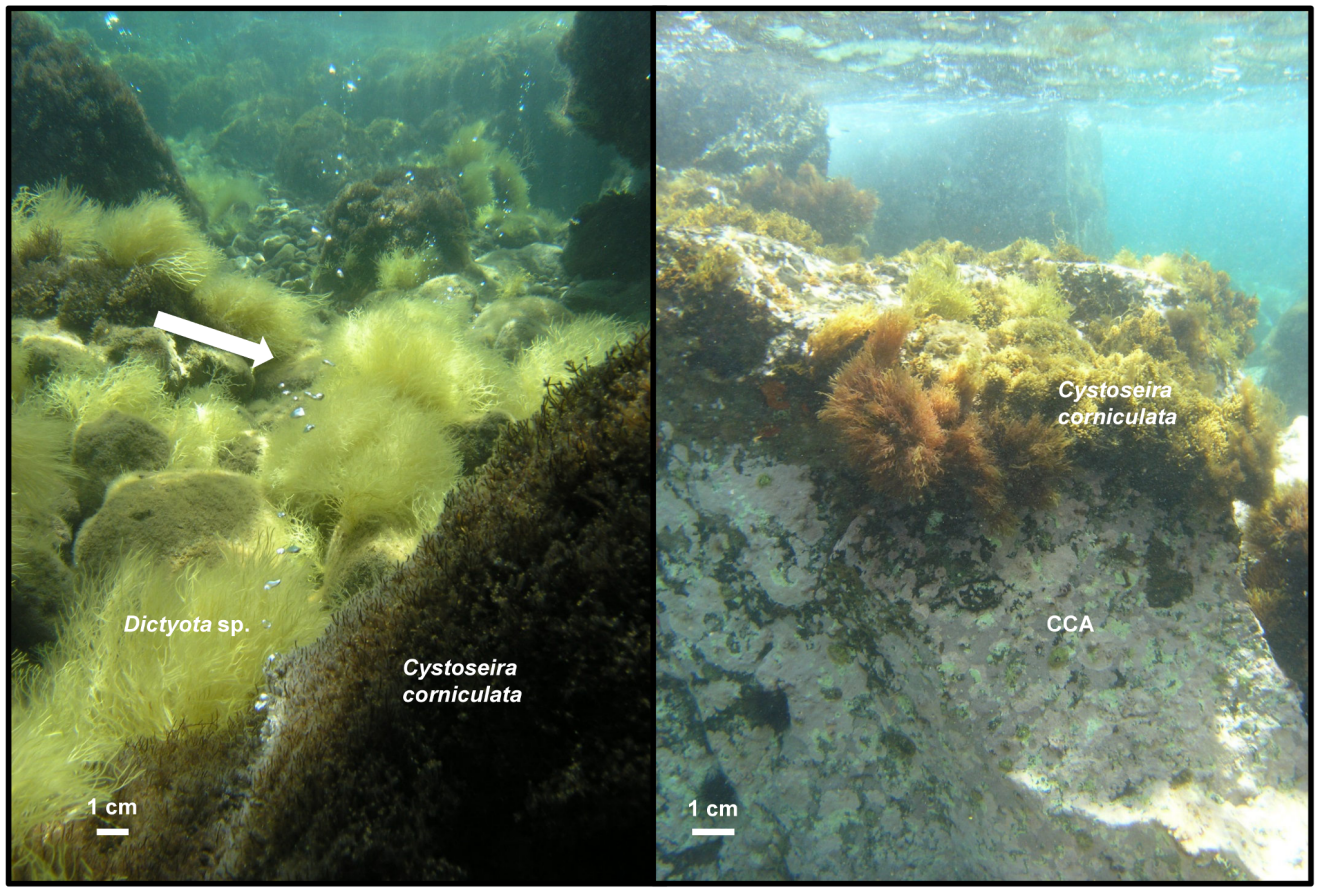
Typical appearance of benthic communities at SEEP (left) and 200 E (right) sites at 0.5 m depth in May 2012 with CO_2_ bubbles seeping from the sea floor (arrow). Brown algae (e.g. *Dictyota* sp.) are dominant near the seeps; crustose coralline algae (CCA) become dominant as CO_2_ levels decrease.

Five sites were selected that had comparable geomorphology and wave exposure, but different pH regimes: a site with pH<8.0 near the main seeps (SEEP), two sites with variable pH located approximately 200 m eastwards and westwards of the seep area (200 E and 200 W) and two reference sites, one just outside the variable pH area (REF A) and one at a more distant site unaffected by volcanic activity (REF B). Wave exposure was estimated using methods in Howes *et al.* [45]. All sites had large boulders and a low degree of urbanisation. Photographs of the typical benthic communities at SEEP and 200 E are shown in Figure 3. The dominant canopy-forming macroalgal species in all sites at <1.5 m depth was *Cystoseira corniculata*, a fucoid alga characteristic of the Eastern Mediterranean Sea [46]. *Cystoseira* spp. are considered indicators of good environmental conditions [47], [48] and *C. corniculata* is common on relatively exposed Eastern Mediterranean rocky shores [49]. No specific permits were required for collecting samples in the present location, as none of the sampling sites are subject to particular protection restrictions, privately-owned or protected in any way; no protected species were sampled in this study.

### Seawater physico-chemical parameters

The seeps were monitored from 2011 to 2013 (September 2011, January, February, May and September 2012, June and September 2013); seawater physicochemical parameters were measured at different times of the day and in different meteorological conditions during each trip. Surface seawater pH, temperature and salinity were measured using a multiprobe (YSI 63). The probe was calibrated before use with pH 4.01, 7.01 and 10.01 NBS standards. Since variations of up to 1 pH unit were detected over a few hours at the high CO_2_ site, the uncertainty in using the NBS scale for seawater pH measurements (approximately 0.05 pH [50]) was considered acceptable for this study. For pH, medians and interquartile ranges (IQ) were calculated from hydrogen ion concentrations before re-converting back to pH values following seep monitoring methods advised by Kerrison *et al.* [14].

Seawater samples for Total Alkalinity (A_T_) determination were collected in 125 ml borosilicate glass bottles with Teflon caps. Three samples per site were collected during each visit, immediately poisoned with HgCl_2_ and stored in the dark until analysis. Samples were analysed by Gran titration (AS-ALK 2, Apollo SciTech) and the reliability of the measurements was checked against standard seawater samples provided by A. Dickson (batch 121). The average A_T_ value per site and individual pH measurements were used to calculate *p*CO_2_, HCO_3_^−^, CO_3_^2−^, Ω_Ar_ and Ω_Ca_ using the CO2SYS software [51].

### Seawater nutrient concentrations

In June 2013 three water samples per site were collected for nutrient analysis. Samples were stored frozen (−20°C), then analysed using a BRAN+LUEBBE II autoanalyser. Inorganic phosphate determination followed the colorimetric method of Murphy and Riley [52] and nitrite ions (NO_2_^−^) were measured colorimetrically according to Bendscheider and Robinson [53]. Determination of nitrate (NO_3_^−^) was performed after its reduction to nitrite, which was then determined colorimetrically as above. Silicate was determined by adding a molybdate solution to the sample. The silicomolybdic acid that formed was then reduced to an intensely blue-coloured complex by adding ascorbic acid as a reducing agent [54]. The determination of ammonium was performed according to Koroleff [55] using a Perkin Elmer 25 Lambda spectrophotometer.

### Free sulphides in seawater

Free sulphides were determined using a method modified from Cline [56]. Three seawater samples per site were collected in May 2012 using plastic syringes, and 2 ml of seawater were injected into a nitrogen-filled septum vial containing a small crystal of cadmium chloride. In order to validate the method, one sample was taken at the sulphide-rich Loutra thermal baths (location shown in Figure 2). For laboratory analysis, most of the water was removed by syringe after allowing the precipitate to settle. The samples were thus reduced to 0.8 ml volume, agitated to suspend all the precipitate and drawn up in a 1 ml disposable syringe which had been flushed with Ar.

Subsequently, 0.2 ml of a solution prepared using 400 mg of N,N-dimethyl-p-phenylene-diamine-dihydrochloride and 600 mg FeCl_3_.6H_2_O dissolved in 100 ml 50% HCl were drawn into the same syringe. The argon bubble in the syringe was used to mix by inverting it a few times. The sample was left to stand for 20 minutes and then injected into a 1 ml semi-microcuvette and read in a Perkin Elmer Lambda 35 UV-VIS spectrometer at 670 nm. Standards were made using a 10 mM sodium sulphide stock solution (249 mg Na_2_S.9 H_2_O in 100 ml degassed Milli-Q water). The stock solution was diluted immediately before use in degassed seawater to give a range of 0.1 to 100 µM.

### Heavy metals in macroalgae

Five individuals of *Dictyota* sp. (Phaeophyta) per site were collected at <2 m depth in May 2012, rinsed with fresh water to eliminate salt, gently brushed to remove epiphytes, kept frozen until transported to the laboratory and then freeze-dried. Freeze-dried macroalgae were ground with pestle and mortar and approximately 0.1 g of each sample was weighed in acid-washed Teflon tubes with a high precision digital scale (0.1 mg accuracy). Two ml of concentrated nitric acid were then added, and the tube containing the digestant was placed in a high-Throughput Microwave Reaction System Run (MARSXpress, CEM Corporation, Matthews, USA) and gently heated to boiling for at least 1 h to ensure full digestion. Samples were allowed to cool and then quantitatively transferred into pre-cleaned 10 ml volumetric flasks and diluted to volume with Milli-Q water. Blanks were prepared following the same procedure, but omitting the sample; a certified reference material (NIES Certified Reference Material No. 3, Chlorella) was simultaneously digested and analysed. Samples were then analysed for heavy metal content (Al, Cd, Cr, Co, Cu, Fe, Pb, Ni, Zn) using inductively coupled plasma optical emission spectrometry ICP-OES) and inductively coupled plasma mass spectrometry (ICP-MS) when concentrations were below the confidence interval of the ICP-OES.

### Benthic communities

Benthic community composition was assessed in May and September 2012: samples were collected from 0.7–1.0 m below mean sea level using 20×20 cm quadrats on sub-horizontal rocky substratum following methods described by Fraschetti *et al.* [57]. A frame with 25 4×4 cm squares was used to assess percentage cover (C%) and number of taxa (S). Percentage cover of algae and sessile invertebrates was determined by assigning each taxon a score ranging from 0 to 4 within each square and summing the 25 estimates following methods described by Dethier *et al.* [58]. Taxa were identified to the lowest possible taxonomic level, usually species. Seven replicate quadrats, randomly chosen but placed at least 4–5 m from each other were assessed for every site in May 2012 and six replicates were collected in September 2012.

### Statistical analyses

Analysis of nutrient and metal concentration data was performed using separate multivariate analyses of variance (MANOVA) with one factor (site). Normality and homogeneity of variances were tested by visually examining boxplots and residual error plots and using Levene's test, and transformed when necessary. When significant differences among sites were detected, a Tukey HSD test for multiple comparisons was performed. Analysis of pH data was performed using a non-parametric analysis (Kruskal-Wallis ANOVA) followed by pairwise multiple comparisons.

Differences in macroalgal community structure and composition were assessed by analysing macroalgal species percent cover with a Permutational Multivariate Analysis of Variance (PRIMER 6 and PERMANOVA + package [59]). The analysis had two fixed factors, season and site. The analysis was performed on Bray-Curtis measures of square-root transformed data, using 9999 permutations of residuals under a reduced model. Pair-wise comparisons were then performed for significant factors with more than two levels. The SIMPER analysis was then used to identify the taxa primarily responsible for the dissimilarity between sites.

Macroalgal cover data were used to calculate Shannon diversity [60] for each sample. The index was analysed using an ANOVA followed by a Tukey HSD test for multiple comparisons. Taxa driving community differences among sites (Table S6 in File S1) were grouped in two categories, canopy-forming algae (*Cystoseira corniculata*, *Cystoseira amentacea*, *Sargassum vulgare* and *Cladostephus spongiosum*) and calcifying algae (CCA, *Jania rubens*, *Corallina* sp., *Amphiroa* sp. and *Padina pavonica*). After testing for normality and homoscedasticity, canopy-forming and calcifying algae arcsin-transformed percent cover was analysed using a two-way ANOVA with site and functional group as fixed factors; seasons were tested separately. The site*functional group interaction was then decomposed to obtain multiple comparisons among sites for each season separately. The same analysis was then performed for selected single species. All univariate analyses were performed using SPSS v19.

## Results

### Seawater physico-chemical parameters

**Figure 4:**
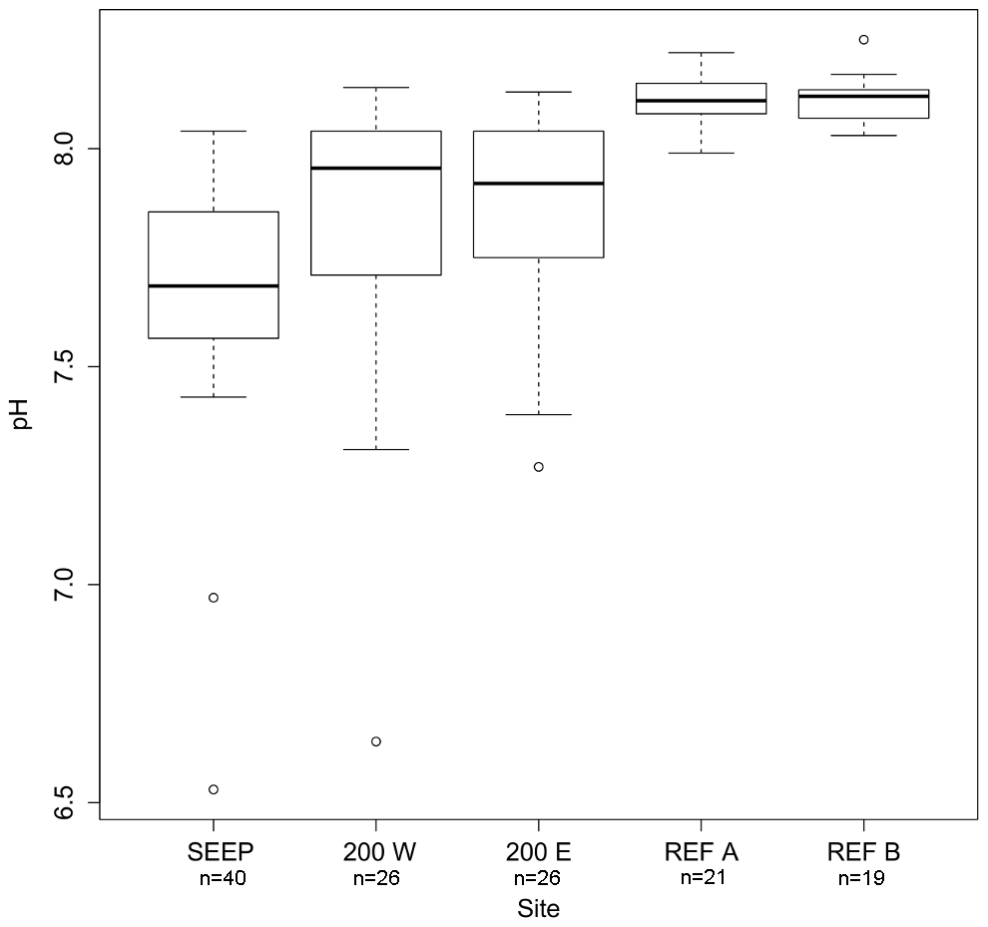
Variability in pH at the five study sites off Methana between September 2011 and September 2013. Horizontal line  =  median, vertical boxes  = 25th and 75th percentiles, whiskers  =  min/max values if smaller than 1.5 times the inter-quartile range and dots  =  outliers.

All sites were classified as semi-exposed according to the classification suggested by Howes *et al.* [45]. Table 2 shows that the seeps had the lowest median pH_NBS_ (7.69, IQ range 7.57–7.85, n = 40) and were significantly different from the intermediate sites, which had higher median values (7.87, n = 26 and 7.96, n = 26 for 200 E and 200 W, respectively; results of statistical analysis shown in Table S1 in File S1) and comparable variability (IQ ranges 7.75–8.04 and 7.73–8.03 for 200 E and 200 W, respectively). At intermediate sites pH sometimes exceeded 8.0. The reference sites had significantly higher pH values (median values of 8.11, n = 21 and 8.12, n = 19 for REF A and REF B, respectively) and lower variability (Figure 4).

**Table 2:** Seawater carbonate chemistry at Methana.

		pH	TA	*p*CO_2_	HCO_3_^−^	CO_3_^2−^		
Site		(NBS)	(mmol/kg)	(µatm)	(mmol/kg)	(mmol/kg)	Ω_Ar_	Ω_Ca_
SEEP	Min	6.53	2.639	24092	2.771	0.006	0.09	0.13
(n_pH_ = 40, n_TA_ = 23)	Median	7.69	2.794	1754	2.538	0.104	1.16	2.45
	Max	7.99	2.944	691	2.243	0.225	3.45	5.20
200 W	Min	6.64	2.696	18652	2.773	0.007	0.11	0.17
(n_pH_ = 26, n_TA_ = 24)	Median	7.96	2.771	872	2.366	0.177	2.70	4.12
	Max	8.14	2.941	526	2.138	0.271	4.18	6.29
200 E	Min	7.27	2.693	4505	2.658	0.038	0.57	0.88
(n_pH_ = 26, n_TA_ = 22)	Median	7.88	2.739	1042	2.403	0.152	2.30	3.50
	Max	8.13	2.836	532	2.114	0.263	4.05	6.10
REF A	Min	7.99	2.640	773	2.261	0.183	2.84	4.30
(n_pH_ = 21, n_TA_ = 18)	Median	8.11	2.708	550	2.106	0.246	3.78	5.70
	Max	8.22	2.769	393	2.049	0.269	4.04	6.18
REF B	Min	8.03	2.615	674	2.254	0.185	2.81	4.30
(n_pH_ = 19, n_TA_ = 15)	Median	8.12	2.697	539	2.145	0.231	3.54	5.33
	Max	8.25	2.858	362	2.024	0.280	4.23	6.46

Measured (pH and total alkalinity) and corresponding calculated carbonate system parameters (*p*CO_2_, bicarbonate and carbonate ions concentrations, saturation state of calcite and aragonite) at five sites using data from six surveys from September 2011 to September 2013. Sample sizes for pH and total alkalinity are shown below site name.

Temperature and salinity varied seasonally and were uniform across sites. The minimum temperature was 14.2°C in February, whereas in summer the temperature could reach 26.8°C; salinity varied from 37.5 to 40.0 ppt. Total Alkalinity varied from 2.615 to 2.944 mmol*kg^−1^ with no seasonal trend (Table 2), with slightly lower values and less variability than CO_2_ vents off Vulcano, where A_T_ varies between 2.78 to 3.17 mmol*kg^−1^ [15]. Seawater *p*CO_2_ had a median value of over 1300 µatm at the SEEP site, almost three times the values calculated for the reference sites. The median saturation state of calcite and aragonite is always >1, although sites with high and intermediate *p*CO_2_ levels were occasionally under-saturated with respect to both calcite and aragonite (Table 2).

Free sulphide concentrations were below the measurable limit (1 µM) for the method used at all five sites. In contrast, our sample from Loutra thermal baths had a concentration of free sulphides of 35 µM. Nutrient concentrations were similar to background levels in the Saronikos Gulf [61] except for silicate, which was mostly higher than the background value of 1.22 µM even at one of the reference sites (Table 3). When values were <LOQ. (Limit Of Quantification) they were substituted with LOQ/2; LOQ. = 0.126 µM for NO_2_+NO_3_ and 0.102 µM for NH_4_. Statistically significant differences between sites were only detected for nitrite and silicate (Table S2 in File S1). Nitrite, however, had a very small range, varying from 0.040±0.005 µM in REF B to 0.054±0.002 µM in 200 E, and these were the only two sites that were significantly different. Silicate had a wider range (from 1.180±0.269 µM in REF B to 6.371±1.841 µM in 200 W); only site 200 W was significantly different from the reference sites according to pairwise comparisons. No significant differences and relatively uniform values were measured for phosphate, whereas nitrate and ammonium showed higher values at 200 E, although these differences were not significant, possibly due to high within-site variability.

**Table 3:** Average seawater nutrient concentrations (±SE, n = 3) at Methana in June 2013.

	SEEP	200 W	200 E	REF A	REF B	Bgd
NO_3_ (µM)	0.070±0.036	0.094±0.040	0.559±0.297	0.054±0.032	0.085±0.026	0.42
NO_2_ (µM)	0.054±0.002^a,b^	0.044±0.003^a,b^	0.059±0.004^b^	0.042±0.002^a,b^	0.040±0.005^a^	n.d.
NH_4_ (µM)	0.232±0.099	0.265±0.109	1.075±0.318	0.203±0.109	0.298±0.053	0.36
PO_4_ (µM)	0.025±0.005	0.031±0.007	0.038±0.009	0.024±0.004	0.044±0	0.12
SiO_4_ (µM)	4.018±0.387^a,b^	6.371±1.841^a^	1.607±0.288^c^	1.883±0.127^b,c^	1.180±0.269^c^	1.22

For the five sites, nitrite, nitrate, ammonium, phosphate and silicate are shown. Background values (Bgd) for the Aegean Sea from Friligos [61]. Different letters indicate significantly different values according to post-hoc pairwise comparisons; n.d.  =  not determined.

### Heavy metals in macroalgae

Measured concentrations of elements in the reference materials were used to assess the quality of the sample measurements; if measured values in the reference material were within 20% of certified values, the quantification of that element was considered reliable. In the reference material analysed, all elements except Pb were within 20% of the certified values, where reported (i.e. excluding Al, Cr, Ni, As). Log-transformed metal concentrations were significantly different between sites for all elements analysed (Table S3 in File S1). Average concentration of elements in *Dictyota* sp. tissues and results of the Tukey HSD test are shown in Table 4. There was a great spatial variability in metal content, but no specific metal concentration consistently increased with decreasing pH. Particularly high concentrations were recorded at station 200 W for aluminium, arsenic and iron, and at REF A for aluminium and zinc.

**Table 4:** *Dictyota* sp. metal content at the five sites.

Element	SEEP	200 W	200 E	REF A	REF B
Al	66.58±29.78^a^	391.84±222.71^b^	75.01±14.21^a,b^	314.62±108.93^a,b^	89.77±17.85^a,b^
As	15.90±1.03^a^	39.02±2.26^d^	25.79±2.68^c^	18.41±1.30^a,b^	22.52±0.37^b,c^
Cd	0.014±0.002^a^	0.018±0.003^a,b^	0.034±0.006^b,c^	0.573±0.102^c^	0.067±0.016^d^
Co	0.059±0.023^a^	0.107±0.020^a^	0.096±0.013^a^	1.613±0.316^b^	0.119±0.016^a^
Cr	0.857±0.070^a,b^	2.526±0.527^c^	0.579±0.050^a^	1.204±0.243^b^	1.093±0.218^a,b^
Cu	2.069±0.228^a^	3.160±0.269^a,b^	3.435±0.569^a,b^	7.726±1.492^c^	4.771±0.303^b,c^
Fe	587.1±42.8^b^	5659.8±603.9^a^	485.5±46.8^b,c^	316.3±88.5^c,d^	146.3±32.5^d^
Ni	0.916±0.100^a^	1.325±0.126^a^	1.338±0.578^a^	4.181±0.267^b^	2.554±0.103^b^
Pb	2.704±0.215^a^	17.605±9.465^b^	2.378±0.276^a^	25.979±11.705^b^	10.820±5.743^b^
Zn	10.95±5.25^a^	11.70±0.53^a^	8.22±0.83^a^	42.02±9.28^b^	14.68±0.60^a,b^

Means (±SE; mg/kg dry weight; n = 5) are shown for each metal and site; different letters indicate significant differences according to Tukey HSD test.

Values higher than ranges reported in the literature for seaweed tissues from unpolluted sites (Table 5) were found for aluminium, arsenic and iron at 200W and for aluminium and zinc in REF A.

**Table 5:** Comparison of metal concentration (mg/kg dry weight) in *Dictyota* spp. measured in this study with values found in the literature for unpolluted sites (n.d.  =  not determined; b.d.l.  =  below detection limit).

	This study	Abdallah *et al.*, 2005 [62]	McDermid and Stuercke, 2003 [63]	Raman *et al.*, 2013 [64]	
Element	(means range)	(mean±SD, n = 3)	(range)	(mean±S.D., n = 3)	Maher and Clarke, 1984 [65]
Al	66–391	n.d.	n.d.	n.d.	n.d.
As	15–39	n.d.	n.d.	n.d.	26.3
Cd	0.014–0.573	0.98±0.3	n.d.	3.9±0.3	n.d.
Co	0.059–1.613	4.3±1.2	n.d.	5.5±0.2	n.d.
Cr	0.579–2.526	1.1±0.3	n.d.	b.d.l.	n.d.
Cu	2–8	1.3±0.4	5	6.4±0.3	n.d.
Fe	316–5659	n.d.	438–608	504±12.4	n.d.
Ni	0.916–4.181	2.2±0.6	n.d.	27±0.4	n.d.
Pb	2–25	19.2±5.5	n.d.	28.5±3.5	n.d.
Zn	8–42	4.9±1.2	13–16	11.7±0.3	n.d.

### Benthic communities

**Figure 5:**
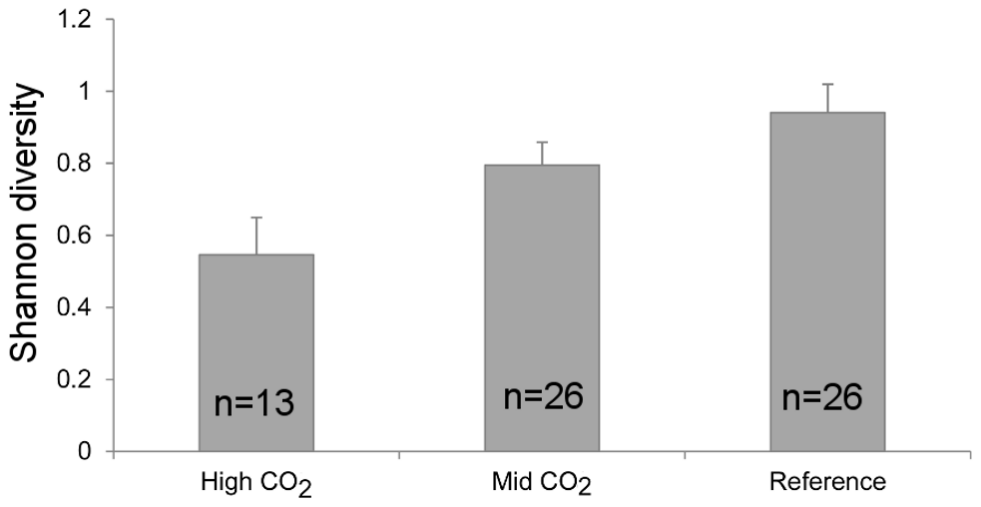
Shannon diversity (mean H' SE) of macroalgal communities at high, intermediate and reference CO_2_ in Methana in May and September 2012.

Overall, 18 macroalgal taxa and three invertebrate taxa (two sponges and one hydrozoan) were recorded. Benthic communities significantly differed among sites and seasons (Table 6), with a significant interaction between the two factors (pseudo-F_4,55_ = 1.754, p(perm) = 0.0457). In spring the high *p*CO_2_ site was significantly different from the reference sites, while the intermediate *p*CO_2_ sites were not significantly different from any of them. In autumn, the high *p*CO_2_ site was significantly different from all other sites (Table 7; results of pairwise comparisons shown in Table S4 in File S1). Site had a significant effect on diversity (p = 0.049, Table S5 in File S1) with a clear decreasing trend as CO_2_ increased, as shown in Figure 5 (0.94±0.10, n = 26 to 0.55±0.08, n = 13; mean ±SE).

**Table 6:** PERMANOVA analyses on square-root transformed percentage cover of Methana benthic communities.

Source	df	SS	Pseudo-F	p (perm)	Unique perms
season	1	31069	19.234	**0.0001**	9949
site	4	21820	3.377	**0.0001**	9918
Season × site	4	11330	1.754	**0.0457**	9916
Residual	55	88840			
Total	64	1.5273E5			

The table shows main factors and their interaction and degrees of freedom (df), sum of squares (SS), pseudo-F, permutational p and unique permutations for each of them.

**Table 7:** Pair-wise comparisons of macroalgal community structure and composition between sites for each season (different letters represent significantly different groups).

Season	Sites				
Spring	SEEP ^a^	200 W ^a,b^	200 E ^a,b^	REF A ^b^	REF B ^b^
Autumn	SEEP ^a^	200 W ^b^	200 E ^b^	REF A ^b^	REF B ^b^

**Figure 6:**
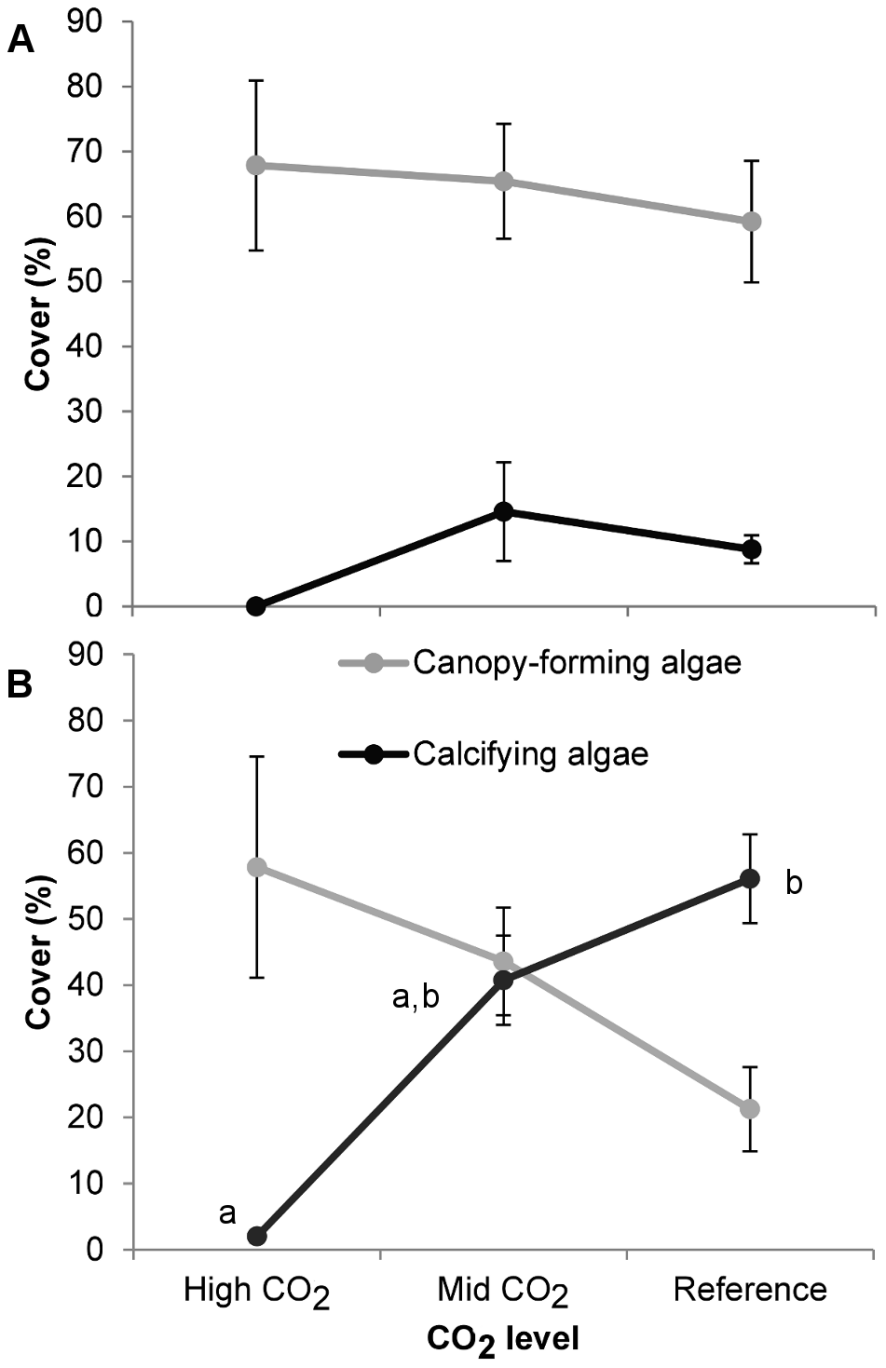
Mean percentage cover (±SE) of canopy-forming algae (grey) and calcifying algae (black) in May (a) and September (b) at high (n = 6), intermediate (n = 14) and reference (n = 14) CO_2_ conditions off Methana. Different letters indicate significant differences between groups.

Percent cover of canopy-forming algae and calcifying algae are shown for May (Figure 6a) and September (Figure 6b). As no significant differences were found within intermediate and reference sites, *p*CO_2_ levels were pooled for clarity. Both categories showed very strong seasonal patterns: no differences in canopy-forming algal cover were detected in May, but in September the high *p*CO_2_ site had a trend towards higher canopy cover compared to the control sites. Likewise, calcifying algae showed no significant difference among *p*CO_2_ levels in spring, but in autumn the high *p*CO_2_ site had a significantly lower cover of calcareous algae compared to intermediate and control *p*CO_2_ levels.

**Figure 7:**
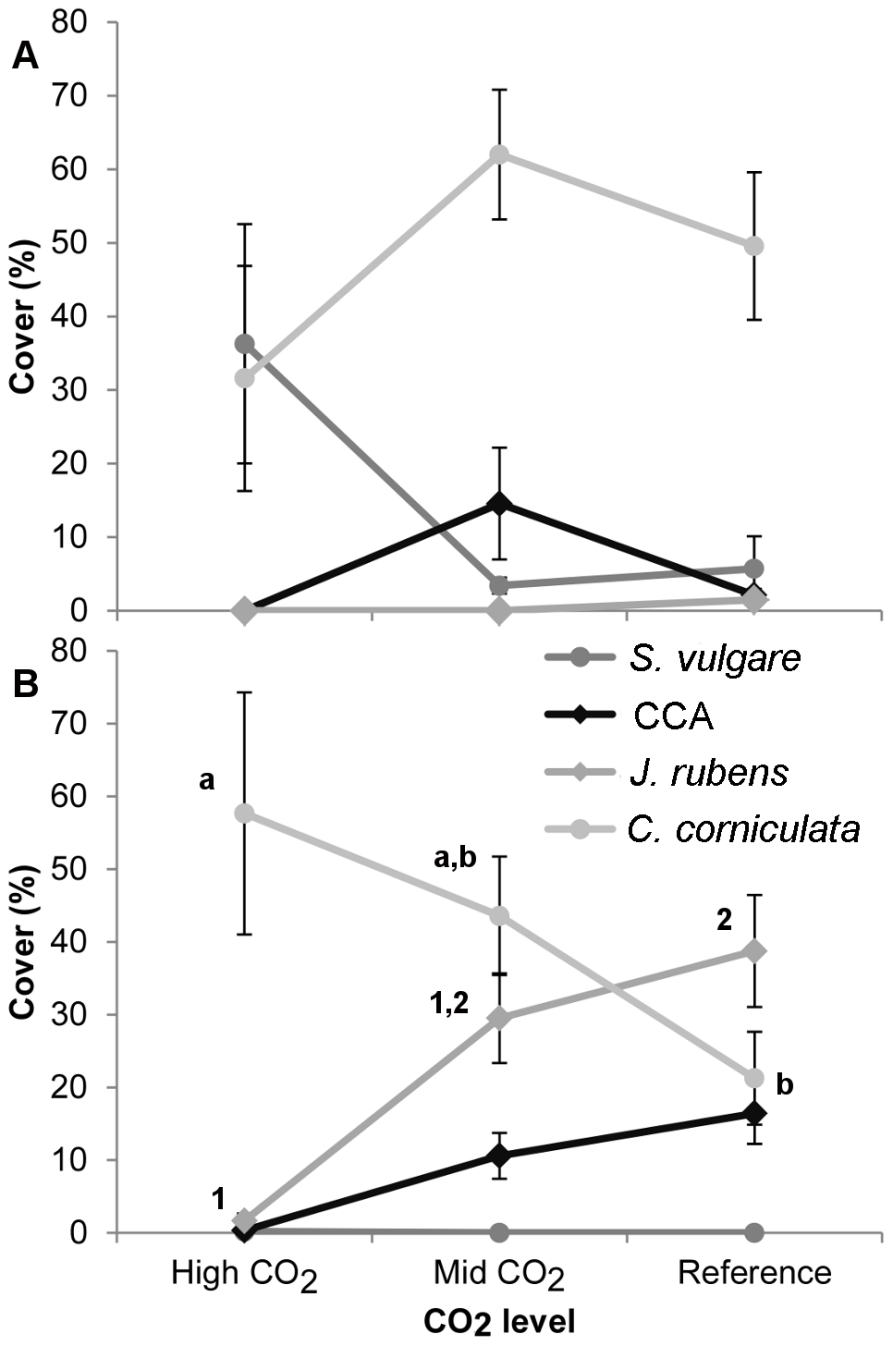
Mean percentage cover (±SE) of dominant macroalgal species in May (a) and September (b) at high (n = 6), intermediate (n = 14) and reference (n = 14) levels of CO_2_ in Methana. Round points represent canopy-forming species (*S. vulgare* dark grey, *C. corniculata* light grey), rhomboids represent calcifying species (CCA black, *J. rubens* grey). Different letters and numbers indicate significant differences between groups.

The species forming these two categories changed along the pCO_2_ gradient depending on the season, and the main canopy-forming and calcareous species covers are shown for May and September in Figure 7a and 7b, respectively. As no significant differences were found within intermediate and reference sites, *p*CO_2_ levels were pooled for clarity. In spring, *S. vulgare* was more abundant at the high *p*CO_2_ site, but it was almost absent from all sites in autumn. In contrast, *C. corniculata* was more constant over time; its cover significantly increased in the high *p*CO_2_ site from spring to autumn, while the opposite was true for the intermediate and reference sites, where *C. corniculata* cover decreased from spring to autumn. As for the coralline algae, CCAs recruited earlier than *J. rubens* and reached their maximum cover in spring at the intermediate sites, while in the reference sites their cover increased from spring to autumn. The articulate coralline alga *J. rubens* had extremely low abundances at all sites in spring, while in autumn its percent cover decreased with increasing *p*CO_2_ levels.

## Discussion

Our results suggest that increased seawater *p*CO_2_ has profound effects on macroalgal communities in oligotrophic conditions, but that sampling season strongly affects the response of benthic communities to ocean acidification. Below we firstly examine the suitability of CO_2_ seeps off Methana for ocean acidification studies, and then discuss the effects of increased carbon dioxide on macroalgal communities.

### Site suitability for ocean acidification studies

Seeps off northern Methana had a median pH value (7.69) similar to that predicted for 2100 according to the IPCC “business as usual” scenario [66], whereas the reference sites had median values above 8. The seeps had no confounding gradients in temperature, salinity, total alkalinity, hydrogen sulphide or wave exposure. The low pH area in Methana had *p*CO_2_ levels comparable to those reported at other ocean acidification analogues [14]–[16], making it suitable to assess community responses to increased *p*CO_2_. Macroalgal community data indicated that elevated carbon dioxide had a profound influence on community composition and structure in an oligotrophic environment, although patterns varied seasonally.

Enrichment in silicate, which was significantly different from reference values in one of the intermediate sites, is likely due to water-rock interactions common in hydrothermal environments [17]. However, it is unlikely that silicate is limiting in the Aegean Sea; for instance, Si becomes limiting to diatoms when the N:Si ratio in seawater is higher than two [67], whereas the background ratio for the Aegean Sea is 0.64 [61]. Significant differences in nitrite concentrations among sites are unlikely to explain the community changes either, as their range is very small (0.040–0.059 µM). Mediterranean organisms are normally not limited by silicate or inorganic nitrogen, but by phosphate [68], for which no confounding gradient was found.

No free sulphides were detected near the seeps, although they were present at the Loutra thermal baths, over 10 km from the study site. Hydrogen sulphide is toxic for cellular respiration, and it is often emitted from Mediterranean volcanic vents [13]. However, sulphides are extremely reactive and oxidise quickly to sulphates in oxygenated waters. It is therefore common to find very low or undetectable sulphide concentrations just a few meters away from volcanic seeps. For instance, at Vulcano sulphides become undetectable at 30 m from the main vents, even though hydrogen sulphide gas has a concentration of 400 ppm at the main bubbling site [15].

Brown algae are a good indicator of bioavailable metal since they are not able to regulate metal uptake [69]. Values higher than ranges reported in the literature were found for aluminium, arsenic and iron at 200 W and for aluminium and zinc in REF A (Table 6). Aluminium variability is likely to be related to local mineralogy [70], while enrichment in the other elements has previously been linked to hydrothermal activity [71]. Metal bioaccumulation is a common occurrence at shallow and deep hydrothermal vents [11], but at Methana metal enrichment did not seem to have major effects at the community and species level. The intermediate and reference sites enriched in some elements (200 W and REF A) were not significantly different from the other intermediate and reference sites (200 E and REF B) with regards to key species percent cover and overall community structure.

The need to translate results from laboratory experiments to more realistic systems has led to several areas with naturally high *p*CO_2_ to be used to infer biological community responses to ocean acidification. Examples include estuaries acidified by acid sulphate soils [72], groundwater submarine springs [73] and upwelling regions [74]. None of the above are perfect ocean acidification analogues, as they can have confounding gradients in salinity and alkalinity (groundwater springs) or in temperature and nutrients (upwelling areas). In addition, low pH recorded in groundwater springs and acidified estuaries is not always caused by increased carbon dioxide concentrations, so only the effects of low pH on biological communities can be tested. However, studies from low pH/high CO_2_ sites mostly report decreased abundance and diversity of calcifying organisms, in accord with findings from CO_2_ seeps and laboratory experiments [2], [9], [10]. General patterns of community responses to ocean acidification can then be detected using areas with naturally low pH, even though confounding factors should always be taken into account.

As with other carbon dioxide seeps used as natural analogues for ocean acidification, Methana has some limitations. Mobile taxa such as fish or some large invertebrates (e.g. cephalopods) are able to move in and out of high CO_2_ areas [75] and pelagic larvae can come from unaffected populations [20]. Moreover, carbonate chemistry is much more variable near the seeps than in reference conditions, as changes in current direction and intensity influence the dispersal of the dissolved gas emissions. Compared to other volcanic seeps, at Methana seawater *p*CO_2_ is high and variable on a greater scale (>15 vs <0.3 km of shoreline [9], [15], [16]). Thus, Methana might offer an opportunity to study ecological processes such as recruitment in a high CO_2_ area probably less influenced by unaffected populations than smaller sites.

### Macroalgal community responses to increased *p*CO_2_

The present study shows that biological responses to elevated carbon dioxide are modulated by season. Macroalgal communities off Methana had year-round decreased diversity, especially of calcifying species, as carbon dioxide increased, in line with results from surveys at other CO_2_ seeps [10], [16], [22] and from laboratory experiments [2], [5]. Seasonality strongly affected community responses to increased *p*CO_2_: coralline algal cover decreased while canopy-forming algae were more abundant as *p*CO_2_ increased, but our sampling design only revealed a significant difference in autumn. This pattern has not been detected so far in macroalgal communities since most field studies have been carried out in one season, while laboratory and mesocosm experiments rarely last long enough to incorporate the effect of seasonality. Godbold and Solan [29] found that seasonality greatly affected invertebrate responses to both ocean acidification and increased temperature.

Our study did not detect an increase in mat-forming algae as CO_2_ increased, in contrast with previous laboratory experiments [36]. However, another shallow subtidal survey off Italian CO_2_ seeps [22] detected a decrease in mat-forming algal biomass at *p*CO_2_ levels of about 1000 ppm. This shows that shifts to mat-forming algae do not necessarily happen at intermediate *p*CO_2_ levels, especially if not associated with increased nutrient levels [36] or other disturbances disrupting kelp cover [76]. In this case, canopy-forming algae appear to increase their growth rates (authors' personal observation), suggesting that macroalgae can use intermediate carbon dioxide levels as a resource [77].

Decreased abundance of calcifying algae is consistent with previous results from volcanic seeps off Ischia, in Italy [22]. However, this pattern was only detected in autumn because of the marked annual cycle of the dominant coralline alga, *Jania rubens*. This species grows best at temperatures above 20°C and reaches its biomass peak later than most other Mediterranean seaweed species [78]. Cover of crustose coralline algae (CCA) decreased as *p*CO_2_ increased, confirming that calcifying algae are likely to be threatened by ocean acidification, especially those species living near their thermal limit [26]. Intermediate *p*CO_2_ levels appeared to increase CCA abundance in spring, possibly because the energy surplus caused by carbon fertilisation is used to enhance calcification when *p*CO_2_ is below 1000 µatm [79], [80]. Recent studies found that CCA are more sensitive to rates, not magnitude, of ocean acidification [81] and that fluctuating pH reduces growth in an articulated coralline alga [4]: high variability in *p*CO_2_ at the seeps could therefore lead to an over-estimation of its negative effects on coralline algae.

The increase in canopy-forming algal cover at high CO_2_ was mostly caused by an increased abundance of *Sargassum vulgare* in spring and of *Cystoseira corniculata* in autumn. *Sargassum vulgare* was more abundant at high CO_2_ also at volcanic seeps off Ischia [22] and Vulcano (authors' personal observation). However, this species was not seen in Methana in autumn because of its pronounced seasonal cycle. As for *C. corniculata*, it is likely that the higher autumnal cover in the elevated *p*CO_2_ site was due to the absence of *S. vulgare* and *J. rubens*. In fact, the genus *Sargassum* can be advantaged over *Cystoseira* when competing for space [82], while *J. rubens* is an epiphyte that can overgrow canopy-forming algae and become dominant in autumn [77]. Physiological responses of *J. rubens* to high *p*CO_2_ are likely to be the main determinant of its decrease in cover, but enhanced chemical defences of *C. corniculata* cannot be excluded, as some fucoid algae are carbon limited, and elevated CO_2_ can cause a sharp increase in their defensive compounds [83].

## Conclusions

Marine volcanic seeps off Methana (Aegean Sea) proved to be suitable for investigations into the response of rocky shore communities to high *p*CO_2_ levels. We found that benthic community changes along *p*CO_2_ gradients in the oligotrophic Mediterranean Sea are consistent across different nutrient regimes. Responses in temperate regions will probably be strongly influenced by seasonality and this alters species interactions during the year. The seeps at Methana revealed loss of diversity and reduced abundance of ecologically important calcifying algae at elevated carbon dioxide levels, adding to a growing body of evidence that ocean acidification is likely to alter coastal community composition [9], [10], [22].

Changes in benthic community structure may have profound effects on biological processes such as food web dynamics, nutrient cycling and primary productivity [84], thus affecting ecosystem functioning. Furthermore, ocean acidification is only one of the many changes marine ecosystems are facing. Additional stressors such as increased temperature or eutrophication are likely to exacerbate the negative effects of increased carbon dioxide [2], [36]. Oligotrophic regions such as the Eastern Mediterranean are therefore extremely vulnerable to future environmental changes, since many organisms already live close to their upper thermal limits, as shown by several mass mortalities following heat waves in recent years [85]. Further research is needed to predict how benthic communities will respond to future environmental conditions, but we provide the first test of subtidal community responses to increased *p*CO_2_ over different seasons and show that seasonal patterns can alter community responses to ocean acidification in warm-temperate coastal ecosystems.

## Supporting Information

File S1 **Supporting tables.** Table S1. Results of the Kruskal-Wallis ANOVA and pairwise comparisons for pH data. Table S2. Effect of site on seawater nutrients as determined by MANOVA. Table S3. Effect of site on seaweed metal concentration as determined by MANOVA. Table S4. PERMANOVA pairwise comparisons of the benthic community structure and composition between sites for each season. Table S5. Effect of site and season on Shannon diversity as determined by ANOVA. Table S6. SIMPER table showing taxa driving difference between sites.
(DOCX)

Data S1 **Carbonate chemistry, nutrient, heavy metal and biological community raw data.**
(XLSX)
