# Supplementary material for: Seasonality Affects Macroalgal Community Response to Increases in pCO2
Source: PLoS One. 2014 Sep 3;9(9):e106520. doi: 10.1371/journal.pone.0106520 (PMC4153631; doi:10.1371/journal.pone.0106520)
Supplement: File S1 — Supporting tables. Table S1. Results of the Kruskal-Wallis ANOVA and pairwise comparisons for pH data. Table S2. Effect of site on seawater nutrients as determined by MANOVA. Table S3. Effect of site on seaweed metal concentration as determined by MANOVA. Table S4. PERMANOVA pairwise comparisons of the benthic community structure and composition between sites for each season. Table S5. Effect of site and season on Shannon diversity as determined by ANOVA. Table S6. SIMPER table showing taxa driving difference between sites. (DOCX) [file pone.0106520.s001.docx]

**Table S1**

| **Total N** | **Test statistics** | **df** | **p** |  |
| --- | --- | --- | --- | --- |
| 132 | 74.886 | 4 | **< 0.001** |  |
|  |  |  |  |  |
| **Site1-Site 2** | **Test statistics** | **Std error** | **Std test statistics** | **p** |
| VENTS-200 E | -27.664 | 9.633 | -2.872 | **0.041** |
| VENTS- 200 W | -27.780 | 9.633 | -2.844 | **0.039** |
| VENTS-REF A | -72.540 | 10.305 | -7.039 | **< 0.001** |
| VENTS-REF B | -73.824 | 10.655 | -6.929 | **< 0.001** |
| 200 E-200 W | -0.115 | 10.606 | -0.011 | 1.000 |
| 200 E-REF A | -44.875 | 11.219 | -4.000 | **0.001** |
| 200 E-REF B | -46.160 | 11.541 | -4.000 | **0.001** |
| 200 W-REF A | -44.760 | 11.219 | -3.990 | **0.001** |
| 200 W-REF B | -46.045 | 11.541 | -3.990 | **0.001** |
| REF A-REF B | -1.284 | 12.108 | -0.106 | 1.000 |

**Table S2**

| **Source** | **Response variable** | **SS** | **df** | **MS** | **F** | **p** |
| --- | --- | --- | --- | --- | --- | --- |
| Site | Ln(NO_3_) | 13.338 | 4 | 3.335 | 3.030 | 0.071 |
|  | Ln(NO_2_) | 0.359 | 4 | 0.090 | 5.851 | **0.011** |
|  | Ln(NH_4_) | 7.624 | 4 | 1.906 | 2.519 | 0.108 |
|  | Ln(PO_4_) | 0.951 | 4 | 0.238 | 2.718 | 0.091 |
|  | Ln(SiO_4_) | 5.584 | 4 | 1.396 | 15.337 | **< 0.001** |
| Error | Ln(NO_3_) | 11.005 | 10 | 1.101 |  |  |
|  | Ln(NO_2_) | 0.153 | 10 | 0.015 |  |  |
|  | Ln(NH_4_) | 7.567 | 10 | 0.757 |  |  |
|  | Ln(PO_4_) | 0.875 | 10 | 0.087 |  |  |
|  | Ln(SiO_4_) | 0.910 | 10 | 0.091 |  |  |
| Corrected Total | Ln(NO_3_) | 24.344 | 14 |  |  |  |
|  | Ln(NO_2_) | 0.513 | 14 |  |  |  |
|  | Ln(NH_4_) | 15.191 | 14 |  |  |  |
|  | Ln(PO_4_) | 1.825 | 14 |  |  |  |
|  | Ln(SiO_4_) | 6.494 | 14 |  |  |  |

**Table S3**

| **Source** | **Response variable** | **Type III SS** | **df** | **MS** | **F** | **p** |
| --- | --- | --- | --- | --- | --- | --- |
| Site | Ln(Al) | 9.164 | 4 | 2.291 | 3.162 | **0.036** |
|  | Ln(Cr) | 5.345 | 4 | 1.336 | 11.656 | **< 0.001** |
|  | Ln(Fe) | 39.322 | 4 | 9.830 | 60.270 | **< 0.001** |
|  | Ln(Co) | 35.888 | 4 | 8.972 | 27.884 | **< 0.001** |
|  | Ln(Ni) | 7.082 | 4 | 1.771 | 25.718 | **< 0.001** |
|  | Ln(Cu) | 4.473 | 4 | 1.118 | 10.114 | **< 0.001** |
|  | Ln(Zn) | 8.218 | 4 | 2.055 | 7.012 | **0.001** |
|  | Ln(As) | 2.392 | 4 | 0.598 | 27.110 | **< 0.001** |
|  | Ln(Cd) | 42.912 | 4 | 10.728 | 62.221 | **< 0.001** |
|  | Ln(Pb) | 46.964 | 4 | 11.741 | 23.247 | **< 0.001** |
| Error | Ln(Al) | 14.490 | 20 | 0.725 |  |  |
|  | Ln(Cr) | 2.293 | 20 | 0.115 |  |  |
|  | Ln(Fe) | 3.262 | 20 | 0.163 |  |  |
|  | Ln(Co) | 6.435 | 20 | 0.322 |  |  |
|  | Ln(Ni) | 1.377 | 20 | 0.069 |  |  |
|  | Ln(Cu) | 2.211 | 20 | 0.111 |  |  |
|  | Ln(Zn) | 5.860 | 20 | 0.293 |  |  |
|  | Ln(As) | 0.441 | 20 | 0.022 |  |  |
|  | Ln(Cd) | 3.448 | 20 | 0.172 |  |  |
|  | Ln(Pb) | 10.101 | 20 | 0.505 |  |  |
| Corrected Total | Ln(Al) | 23.655 | 24 |  |  |  |
|  | Ln(Cr) | 7.637 | 24 |  |  |  |
|  | Ln(Fe) | 42.584 | 24 |  |  |  |
|  | Ln(Co) | 42.324 | 24 |  |  |  |
|  | Ln(Ni) | 8.459 | 24 |  |  |  |
|  | Ln(Cu) | 6.684 | 24 |  |  |  |
|  | Ln(Zn) | 14.079 | 24 |  |  |  |
|  | Ln(As) | 2.834 | 24 |  |  |  |
|  | Ln(Cd) | 46.360 | 24 |  |  |  |
|  | Ln(Pb) | 57.065 | 24 |  |  |  |

**Table S4**

| **Spring** | | | |
| --- | --- | --- | --- |
| **Site1, Site 2** | **t** | **P(perm)** | **Unique perms** |
| SEEP, 200 E | 1.403 | 0.115 | 1709 |
| SEEP, 200 W | 1.595 | 0.075 | 1711 |
| SEEP, REF A | 1.771 | **0.031** | 1706 |
| SEEP, REF B | 1.675 | **0.048** | 1704 |
| 200 E, 200 W | 0.587 | 0.738 | 1709 |
| 200 E, REF A | 1.470 | 0.088 | 1708 |
| 200 E, REF B | 1.218 | 0.202 | 1708 |
| 200 W, REF A | 1.518 | 0.079 | 1707 |
| 200 W, REF B | 1.426 | 0.088 | 1709 |
| REF A, REF B | 0.955 | 0.452 | 1704 |
| **Autumn** | | | |
| **Site1, Site 2** | **t** | **P(perm)** | **perms** |
| SEEP, 200 E | 2.707 | **0.002** | 336 |
| SEEP, 200 W | 1.677 | **0.037** | 462 |
| SEEP, REF A | 2.965 | **0.002** | 462 |
| SEEP, REF B | 1.812 | **0.032** | 462 |
| 200 E, 200 W | 1.225 | 0.242 | 336 |
| 200 E, REF A | 1.097 | 0.272 | 336 |
| 200 E, REF B | 1.385 | 0.134 | 336 |
| 200 W, REF A | 1.320 | 0.170 | 462 |
| 200 W, REF B | 1.024 | 0.376 | 462 |
| REF A, REF B | 1.173 | 0.276 | 462 |

**Table S5**

| **Source** | **Type III SS** | **df** | **MS** | **F** | **p** |
| --- | --- | --- | --- | --- | --- |
| Site | 1.438 | 4 | 0.359 | 2.557 | **0.049** |
| Season | 0.261 | 1 | 0.261 | 1.859 | 0.178 |
| Site * Season | 0.232 | 4 | 0.058 | 0.413 | 0.798 |
| Error | 7.731 | 55 | 0.141 |  |  |
| Total | 51.627 | 65 |  |  |  |
| Corrected Total | 9.664 | 64 |  |  |  |

**Table S6**

| **Groups SEEP & 200 E; Average dissimilarity = 65.40** | | | | | | |
| --- | --- | --- | --- | --- | --- | --- |
|  | SEEP | 200 E |  |  |  |  |
| Taxon | Av.Abund | Av.Abund | Av.Diss | Diss/SD | Contrib% | Cum.% |
| *Cystoseira corniculata* | 5.27 | 6.76 | 14.85 | 1.28 | 22.71 | 22.71 |
| CCA | 0.11 | 2.86 | 8.8 | 0.88 | 13.45 | 36.16 |
| *Sargassum vulgare* | 2.58 | 0.85 | 8.46 | 0.74 | 12.93 | 49.09 |
| *Jania rubens* | 0.00 | 2.63 | 7.7 | 0.78 | 11.77 | 60.85 |
| *Dictyota* sp. | 1.16 | 1.59 | 6.14 | 0.91 | 9.38 | 70.24 |
| *Sargassum* sp. | 1.50 | 0.00 | 4.28 | 0.45 | 6.54 | 76.78 |
| *Padina pavonica* (turfing form) | 1.12 | 0.27 | 3.93 | 0.51 | 6.02 | 82.79 |
| Bare substratum | 1.08 | 0.00 | 3.16 | 0.60 | 4.83 | 87.62 |
| *Falkenbergia* sp. | 0.46 | 0.00 | 1.38 | 0.51 | 2.10 | 89.73 |
| *Cladophora* sp. | 0.50 | 0.00 | 1.36 | 0.39 | 2.08 | 91.80 |
| **Groups SEEP & 200 W; Average dissimilarity = 63.83** | | | | | | |
|  | SEEP | 200 W |  |  |  |  |
| Taxon | Av.Abund | Av.Abund | Av.Diss | Diss/SD | Contrib% | Cum.% |
| *Cystoseira corniculata* | 5.27 | 6.52 | 14.63 | 1.30 | 22.92 | 22.92 |
| *Sargassum vulgare* | 2.58 | 0.59 | 8.96 | 0.70 | 14.04 | 36.96 |
| *Cladostephus spongiosus* | 0.36 | 1.95 | 6.54 | 0.85 | 10.24 | 47.20 |
| *Dictyota* sp. | 1.16 | 1.56 | 6.18 | 0.85 | 9.68 | 56.88 |
| *Jania rubens* | 0.00 | 1.90 | 5.46 | 0.65 | 8.56 | 65.44 |
| CCA | 0.11 | 1.57 | 4.81 | 0.63 | 7.53 | 72.97 |
| *Sargassum* sp. | 1.50 | 0.55 | 4.27 | 0.52 | 6.69 | 79.65 |
| *Padina pavonica* (turfing form) | 1.12 | 0.26 | 3.96 | 0.51 | 6.21 | 85.86 |
| Bare substratum | 1.08 | 0.64 | 2.89 | 0.58 | 4.53 | 90.39 |
| **Groups 200 E & 200 W; Average dissimilarity = 43.27** | | | | | | |
|  | 200 E | 200 W |  |  |  |  |
| Taxon | Av.Abund | Av.Abund | Av.Diss | Diss/SD | Contrib% | Cum.% |
| *Cystoseira corniculata* | 6.76 | 6.52 | 9.58 | 1.11 | 22.14 | 22.14 |
| CCA | 2.86 | 1.57 | 8.68 | 0.92 | 20.06 | 42.21 |
| *Cladostephus spongiosus* | 0.00 | 1.95 | 5.86 | 0.82 | 13.54 | 55.74 |
| *Jania rubens* | 2.63 | 1.90 | 3.41 | 0.60 | 7.87 | 63.61 |
| *Dictyota* sp. | 1.59 | 1.56 | 3.11 | 0.66 | 7.18 | 70.80 |
| *Sargassum vulgare* | 0.85 | 0.59 | 2.81 | 0.74 | 6.50 | 77.30 |
| Bare substratum | 0.00 | 0.64 | 1.62 | 0.40 | 3.75 | 81.05 |
| *Padina pavonica* (turfing form) | 0.27 | 0.26 | 1.54 | 0.40 | 3.56 | 84.61 |
| *Halopteris scoparia* | 0.15 | 0.42 | 1.54 | 0.39 | 3.56 | 88.17 |
| *Sargassum* sp. | 0.00 | 0.55 | 1.44 | 0.49 | 3.33 | 91.49 |
| **Groups SEEP & REF A; Average dissimilarity = 74.58** | | | | | | |
|  | SEEP | REF A |  |  |  |  |
| Taxon | Av.Abund | Av.Abund | Av.Diss | Diss/SD | Contrib% | Cum.% |
| *Cystoseira corniculata* | 5.27 | 5.01 | 15.26 | 1.36 | 20.46 | 20.46 |
| *Jania rubens* | 0.00 | 3.77 | 11.28 | 0.94 | 15.13 | 35.59 |
| *Sargassum vulgare* | 2.58 | 0.34 | 8.76 | 0.70 | 11.75 | 47.34 |
| Bare substratum | 1.08 | 1.71 | 6.83 | 1.29 | 9.15 | 56.50 |
| *Dictyota* sp. | 1.16 | 1.98 | 6.49 | 0.76 | 8.70 | 65.20 |
| CCA | 0.11 | 1.83 | 5.13 | 0.87 | 6.87 | 72.07 |
| *Sargassum* sp. | 1.50 | 0.00 | 4.28 | 0.45 | 5.74 | 77.81 |
| *Padina pavonica* (turfing form) | 1.12 | 0.00 | 3.27 | 0.44 | 4.38 | 82.19 |
| *Cystoseira amentacea* | 0.00 | 0.93 | 2.93 | 0.53 | 3.93 | 86.12 |
| *Padina pavonica* (calcified form) | 0.00 | 0.75 | 2.44 | 0.52 | 3.27 | 89.40 |
| *Halopteris scoparia* | 0.00 | 0.66 | 2.03 | 0.40 | 2.73 | 92.12 |
| **Groups 200 E & REF A; Average dissimilarity = 46.56** | | | | | | |
|  | 200 E | REF A |  |  |  |  |
| Taxon | Av.Abund | Av.Abund | Av.Diss | Diss/SD | Contrib% | Cum.% |
| *Cystoseira corniculata* | 6.76 | 5.01 | 11.28 | 1.21 | 24.22 | 24.22 |
| CCA | 2.86 | 1.83 | 6.48 | 0.78 | 13.93 | 38.15 |
| Bare substratum | 0.00 | 1.71 | 5.02 | 0.99 | 10.77 | 48.92 |
| *Dictyota* sp. | 1.59 | 1.98 | 4.84 | 0.84 | 10.39 | 59.31 |
| *Jania rubens* | 2.63 | 3.77 | 4.39 | 0.86 | 9.43 | 68.74 |
| *Sargassum vulgare* | 0.85 | 0.34 | 3.15 | 0.77 | 6.77 | 75.50 |
| *Cystoseira amentacea* | 0.00 | 0.93 | 2.81 | 0.53 | 6.04 | 81.55 |
| *Padina pavonica* (calcified form) | 0.00 | 0.75 | 2.34 | 0.52 | 5.04 | 86.58 |
| *Halopteris scoparia* | 0.15 | 0.66 | 2.30 | 0.48 | 4.95 | 91.53 |
| **Groups 200 W & REF A; Average dissimilarity = 53.21** | | | | | | |
|  | 200 W | REF A |  |  |  |  |
| Taxon | Av.Abund | Av.Abund | Av.Diss | Diss/SD | Contrib% | Cum.% |
| *Cystoseira corniculata* | 6.52 | 5.01 | 10.33 | 1.20 | 19.42 | 19.42 |
| CCA | 1.57 | 1.83 | 6.12 | 0.92 | 11.50 | 30.92 |
| Bare substratum | 0.64 | 1.71 | 5.85 | 1.14 | 10.99 | 41.91 |
| *Jania rubens* | 1.90 | 3.77 | 5.74 | 0.80 | 10.78 | 52.69 |
| *Cladostephus spongiosus* | 1.95 | 0.00 | 5.66 | 0.81 | 10.64 | 63.33 |
| *Dictyota* sp. | 1.56 | 1.98 | 4.96 | 0.78 | 9.32 | 72.65 |
| *Cystoseira amentacea* | 0.00 | 0.93 | 2.82 | 0.53 | 5.31 | 77.96 |
| *Halopteris scoparia* | 0.42 | 0.66 | 2.56 | 0.48 | 4.82 | 82.77 |
| *Sargassum vulgare* | 0.59 | 0.34 | 2.40 | 0.58 | 4.51 | 87.29 |
| *Padina pavonica* (calcified form) | 0.00 | 0.75 | 2.35 | 0.52 | 4.42 | 91.71 |
| **Groups SEEP & REF B; Average dissimilarity = 72.33** | | | | | | |
|  | SEEP | REF B |  |  |  |  |
| Taxon | Av.Abund | Av.Abund | Av.Diss | Diss/SD | Contrib% | Cum.% |
| *Cystoseira corniculata* | 5.27 | 4.64 | 15.07 | 1.30 | 20.84 | 20.84 |
| *Sargassum vulgare* | 2.58 | 0.60 | 8.90 | 0.71 | 12.31 | 33.15 |
| *Sargassum* sp. | 1.50 | 1.86 | 7.36 | 0.81 | 10.18 | 43.33 |
| CCA | 0.11 | 2.44 | 6.80 | 1.15 | 9.41 | 52.73 |
| *Jania rubens* | 0.00 | 2.09 | 5.96 | 0.79 | 8.24 | 60.97 |
| *Dictyota* sp. | 1.16 | 1.61 | 5.76 | 0.76 | 7.97 | 68.94 |
| Bare substratum | 1.08 | 1.46 | 4.65 | 0.88 | 6.42 | 75.36 |
| *Padina pavonica* (calcified form) | 0.00 | 1.18 | 3.66 | 0.87 | 5.06 | 80.42 |
| *Padina pavonica* (turfing form) | 1.12 | 0.00 | 3.26 | 0.44 | 4.51 | 84.92 |
| *Halopteris scoparia* | 0.00 | 0.97 | 2.98 | 0.43 | 4.12 | 89.04 |
| *Falkenbergia* sp. | 0.46 | 0.00 | 1.32 | 0.51 | 1.82 | 90.86 |
| **Groups 200 E & REF B; Average dissimilarity = 53.38** | | | | | | |
|  | 200 E | REF B |  |  |  |  |
| Taxon | Av.Abund | Av.Abund | Av.Diss | Diss/SD | Contrib% | Cum.% |
| *Cystoseira corniculata* | 6.76 | 4.64 | 12.03 | 1.18 | 22.55 | 22.55 |
| CCA | 2.86 | 2.44 | 7.52 | 1.06 | 14.08 | 36.62 |
| *Sargassum* sp. | 0.00 | 1.86 | 5.37 | 0.69 | 10.06 | 46.69 |
| *Jania rubens* | 2.63 | 2.09 | 4.08 | 0.76 | 7.64 | 54.33 |
| Bare substratum | 0.00 | 1.46 | 4.00 | 0.70 | 7.49 | 61.81 |
| *Sargassum vulgare* | 0.85 | 0.60 | 3.96 | 0.68 | 7.42 | 69.23 |
| *Dictyota* sp. | 1.59 | 1.61 | 3.96 | 0.79 | 7.41 | 76.64 |
| *Padina pavonica* (calcified form) | 0.00 | 1.18 | 3.50 | 0.87 | 6.56 | 83.20 |
| *Halopteris scoparia* | 0.15 | 0.97 | 3.12 | 0.49 | 5.84 | 89.04 |
| *Padina pavonica* (turfing form) | 0.27 | 0.00 | 0.83 | 0.30 | 1.55 | 90.59 |
| **Groups 200 W & REF B; Average dissimilarity = 57.06** | | | | | | |
|  | 200 W | REF B |  |  |  |  |
| Taxon | Av.Abund | Av.Abund | Av.Diss | Diss/SD | Contrib% | Cum.% |
| *Cystoseira corniculata* | 6.52 | 4.64 | 11.48 | 1.23 | 20.13 | 20.13 |
| CCA | 1.57 | 2.44 | 6.89 | 1.09 | 12.07 | 32.20 |
| *Cladostephus spongiosus* | 1.95 | 0.00 | 5.55 | 0.82 | 9.73 | 41.93 |
| *Sargassum* sp. | 0.55 | 1.86 | 5.48 | 0.80 | 9.60 | 51.54 |
| Bare substratum | 0.64 | 1.46 | 4.21 | 0.78 | 7.38 | 58.92 |
| *Dictyota* sp. | 1.56 | 1.61 | 4.17 | 0.76 | 7.32 | 66.24 |
| *Jania rubens* | 1.90 | 2.09 | 4.04 | 0.75 | 7.09 | 73.32 |
| *Halopteris scoparia* | 0.42 | 0.97 | 3.83 | 0.54 | 6.71 | 80.03 |
| *Padina pavonica* (calcified form) | 0.00 | 1.18 | 3.49 | 0.87 | 6.12 | 86.15 |
| *Sargassum vulgare* | 0.59 | 0.60 | 3.22 | 0.52 | 5.64 | 91.79 |
| **Groups REF A & REF B; Average dissimilarity = 54.82** | | | | | | |
|  | REF A | REF B |  |  |  |  |
| Taxon | Av.Abund | Av.Abund | Av.Diss | Diss/SD | Contrib% | Cum.% |
| *Cystoseira corniculata* | 5.01 | 4.64 | 10.88 | 1.19 | 19.84 | 19.84 |
| *Jania rubens* | 3.77 | 2.09 | 6.05 | 0.89 | 11.03 | 30.87 |
| Bare substratum | 1.71 | 1.46 | 6.03 | 1.13 | 10.99 | 41.86 |
| *Sargassum* sp. | 0.00 | 1.86 | 5.24 | 0.68 | 9.55 | 51.41 |
| *Dictyota* sp. | 1.98 | 1.61 | 4.95 | 0.75 | 9.03 | 60.44 |
| CCA | 1.83 | 2.44 | 4.91 | 1.23 | 8.95 | 69.39 |
| *Halopteris scoparia* | 0.66 | 0.97 | 4.17 | 0.61 | 7.60 | 76.99 |
| *Padina pavonica* (calcified form) | 0.75 | 1.18 | 3.78 | 0.93 | 6.89 | 83.88 |
| *Cystoseira amentacea* | 0.93 | 0.00 | 2.65 | 0.53 | 4.84 | 88.71 |
| *Sargassum vulgare* | 0.34 | 0.60 | 2.58 | 0.40 | 4.70 | 93.42 |
|  |  |  |  |  |  |  |
